# The Effect of Mid-to-Long-Term Hospitalization on the Catastrophic Health Expenditure: Focusing on the Mediating Effect of Earned Income Loss

**DOI:** 10.3390/healthcare9081013

**Published:** 2021-08-07

**Authors:** Hyunwoo Jung, Jungyeon Yang, Eunyoung Kim, Junhyup Lee

**Affiliations:** 1 Department of Health Administration, Graduate School, Yonsei University, Wonju 26493, Korea; jhw8901@naver.com; 2 Transdisciplinary Major in Learning Health Systems, Department of Public Health Science, Graduate School, Korea University, Seoul 02841, Korea; jyyang0705@korea.ac.kr; 3 Department of Biomedical Laboratory Science, Songho University, Hoengseong 25242, Korea; keysr@hanmail.net; 4 Department of Public Health Science, Korea University, Seoul 02841, Korea

**Keywords:** hospitalization, catastrophic health expenditure, income loss

## Abstract

Background: Mid-to-long-term hospitalization (MLTH) can threaten the household economy with high medical costs and loss of income. Therefore, it could increase the catastrophic health expenditure (CHE), measured as the ratio of medical expenses to the ability to pay. This study aimed to determine the effect of MLTH on the incidence of CHE and the mediating effect of earned income reduction rate (EIRR). Methods: We used 2015 to 2017 data from the Korean Welfare Panel Study and selected households with earned income through work. The final samples were 1671 households in the database. This study applied three-step regression analyses for estimating mediation effects. Results: First, MLTH affected CHE increases; second, MLTH increased EIRR; third, both EIRR and MLTH increased CHE at the same time. Additionally, the bootstrapping results were 0.364 to 0.644 in the 95% confidence interval, which suggested that EIRR mediated the effects of MLTH on CHE. Conclusions: Previous studies have only focused on medical costs when interpreting CHE; however, it is also essential to recognize that the MLTH can have a negative effect on the EIRR. This study contributed to the literature by giving another insight into interpreting the cause of CHE, focusing on income loss factors.

## 1. Introduction

Medical events can lead not only to medical expenses but also to income loss due to suspension of work activities [1,2,3]. Loss of earned income can create a greater economic burden than short-term medical expenses. Although other types of income, such as private transfer income, can resolve short-term medical expenses, reduced earned income can continue to harm the household financially.

Many high-income countries already provide sickness benefits to reduce income loss. In Germany, for example, if a worker is unable to work due to sickness, 100% of the current salary is guaranteed for the first 6 weeks, and 70−90% for up to 78 weeks thereafter [4,5]. In Sweden, from the 14 days after the occurrence of an injury, the employer guarantees 100% of the wages to the worker, and from the 14th to up to the 550th day, social insurance guarantees the worker 80% of the existing wage [4,6]. However, Korea, even now classified as a high-income country, does not include sickness benefits in the public social security system [7].

Moreover, the United Nations had chosen the financial risk protection indicator (3.8.2 indicator) for Universal Health Coverage in the declaration of 2015 sustainable development goals. This indicator states, “Proportion of population with large household expenditures on health as a share of total household expenditure or income” [8], which adopted the concept and definition of catastrophic health expenditure (CHE) suggested by Xu et al. [9] and Wagstaff and van Doorslaer [10]. They defined “catastrophic” when households spend more than a certain percentage of their ability to pay. The ability to pay means the effective income remaining after basic subsistence needs (e.g., food expenses).

Since the CHE consists of two variables (medical expenses and the household’s ability to pay), both sides are equally important; however, most studies that examine CHE only focus on the issue of medical expenses [11,12,13,14]. If income declines, the risk of CHE becomes more severe even with the same medical expenses. Therefore, focusing solely on the medical expenses can be a limited approach.

In this regard, mid-to-long-term hospitalization (MLTH) is a representative health issue that could produce medical expenses and income loss. Hospitalization itself means incurring medical expenses and also implies that the degree of deterioration in health is more severe than that of other medical uses such as outpatient treatment and medication prescription. Furthermore, since the patient stays in the hospital, they temporarily stop working. MLTH needs specialized medical treatments and a longer recovery period even after discharge so that the patient would not be able to engage in work activities for a certain period of time after hospitalization. 

This study aimed to analyze the effect of MLTH on the incidence of CHE and the mediating effect of loss of earned income. Therefore, this study considered the earned income reduction rate (EIRR) before and after hospitalization, conducting the three-step mediating effect analysis by setting MLTH as an independent variable, CHE as a dependent variable, and the EIRR as a parameter. Our research hypotheses were as follows: (1) MLTH affects CHE increase. (2) MLTH increases EIRR. (3) EIRR mediates the effect of MLTH on CHE. Finally, this paper suggests the implementation of a guarantee system against income loss due to poor health.

## 2. Methods

### 2.1. Data Source and Research Subjects

This study utilized data derived from Korea Welfare Panel Study (KoWePS), nationally representative data which includes the regionally remote countryside. The KoWePS was administered by the Korea Institute of Health and Social Affairs in conjunction with the Seoul National University in South Korea. The samples of KoWePS were recruited using a dual sampling frame of the national population and housing census and a combination of random digit dial and address-based sampling. It provides information on socio-demographic information, medical expenses, and income at the individual and household level. Additionally, to alleviate the recall bias problem, it used household financial record books or medical expenses receipts and arranged the investigation’s timing of March to May, which is the report time of the comprehensive income tax return. Originally, KoWePS started with 7072 household participants in 2005, though as time goes by, the original sample’s retention rate was 64.48%, namely 4560 households in 2017, because of the nature of the panel survey. This study analyzed the effect of MLTH on CHE using KoWePS data from 2015, 2016, and 2017. Only those households who responded to the 2015, 2016, and 2017 surveys, whose earned income had decreased since 2015 were included in the study. Finally, 1671 households without missing values of major variables were selected as the target of analysis. This study was approved by the Institutional Review Board (IRB) of Korea University.

### 2.2. Statistical Analysis

#### 2.2.1. Measurement of Variables

The dependent variable was whether CHE was incurred in 2017. The independent variable was the MLTH in 2016. The parameter was the EIRR between 2015 and 2017. The dependent variable, CHE, was calculated according to the method proposed by Wagstaff and van Doorslaer [10]. Household ability to pay was measured by excluding the expense of actually consumed food from household total income, and based on this, the medical expenses ratio was measured. The variables of health care expenses of the KoWePS include the cost of hospitalization, outpatient care, dental treatment, surgery, medicines, medical care, postpartum care, health checkups, health supplements, medical supplies, and the use of treatment vouchers. The threshold of CHE was set at 10%, in accordance with recent studies [13,15,16].

MLTH was defined as hospitalization more than 6 days in 2016 and was measured for each household. Using data from the Korea Institute for Health and Social Affairs on length of stays in hospital and considering the distribution of inpatients, Yoon [17] classified 1 week of hospitalization as short-term hospitalization, 8 days or more and 179 days or less as mid-term hospitalization, and over 180 days as long-term hospitalization.

The present study also classified MLTH by considering the distribution of inpatients in the 2016 data from KoWePS. In the examination of the distribution of the length of stay, it was observed that the number of patients tended to decrease as the length of stay increased. However, the number of patients continued to decrease until the sixth day, and then the number of patients increased sharply again from the seventh day, which was the highest among all lengths of stay. After the seventh day, it decreased significantly again. If MLTH had been defined for more than 8 days, then the sample would have been small; therefore, it was set to 7 days or more.

EIRR was calculated as follows: (2015 income–2017 income)/2015 income. Because the level of earned income differs exponentially from household to household, it is necessary to convert it into a form of normal distribution that allows for regression analysis [18]. Therefore, what was calculated by taking the natural logarithm of the decrease in earned income was measured as a variable for the EIRR.

In addition, this study included covariates to control other factors. Firstly, as householder’s characteristics, gender, age, education level, marital status, and job type were included. Secondly, the number of household members, medical coverage type, private health insurance, the existence of chronic disease, and disability were included as household characteristics.

#### 2.2.2. Mediating Effect Test

The mediating effect model of Baron and Kenny [19] was used to verify the mediating effect. It consists of three-step regression equations. In Model 1, it is necessary to check whether the independent variable has a significant effect on the dependent variable with the parameter excluded from the model. In Model 2, parameters are set as dependent variables to check whether independent variables have an effect. Finally, in Model 3, the influence of the independent variable and the parameter on the dependent variable is checked by simultaneously inserting it into the model. 

According to the Model 3 results, mediating effects are classified as two types as follows: If both the independent variable and the parameter are significant, it is considered that there is a partial mediating effect. This means that the independent variable and the parameter individually affect the dependent variable. Otherwise, if only the parameters are significant, it means that the independent variable can affect the dependent variable only through the parameter. This is called the complete mediating effect.

At this time, the parameter must be statistically significant. If a parameter is significant, it can be considered to have a mediating effect whether the independent variable is significant or not. In general, a mediating effect model is set based on a continuous variable [20], but in this study, since a mediating effect model including a binary dependent variable is assumed, in order to avoid problems [21], such as a violation of the normality of errors, a violation of equal variance, and a biased standard error, standardized regression coefficients and logistic regression models were used [22,23,24].

In this study, the effect of MLTH on CHE was analyzed by multivariate logit regression analysis in Model 1, and the effect of MLTH on earned income was analyzed by linear regression analysis in Model 2. In Model 3, a multivariate logit regression analysis was performed by simultaneously inputting MLTH and earned income into the model. All analyses included the sociodemographic characteristics of households and householders as covariates. Finally, in the step of verifying whether there is a mediating effect, a bootstrapping test was performed. In bootstrapping, if 0 is not included between the upper and lower limits of the 95% confidence interval, the mediating effect is considered to be significant.

Regression coefficients of all the steps in the analysis were converted to standardized regression coefficient values (beta). All analyses were performed using STATA version 14 (StataCorp, College Station, TX, USA).

## 3. Results

### 3.1. General Characteristics of the Study Subjects

The general characteristics of the study subjects are described in Table 1. Among all the householders, 76.1% were men and 23.8% were women, which was similar to the men-to-women ratio of householders included in the KoWePS. In terms of age, 31.7% of the subjects were aged 39 years or younger, 52.7% of the subjects were aged 40 to 65 years, and 15.4% were aged 65 years or older. As for marital status, 66.1% were married; 5.6% were single; and 28.1% were divorced, widowed, or separated. In terms of education level, the majority had an elementary school or below level of education (47.7%). Job types included wage worker (46.9%), employers or self-employed (20.7%), unpaid family worker (2.9), and unemployed (29.3%). As for the number of households, two-person households were the most common (33.2%), followed by four-person households (24.9%). In the type of medical coverage, employees was the most common at 64.3%, followed by employer or self-employed (29.9). Among all the subjects, 4.9% were disabled, and 72.2% had chronic diseases.

### 3.2. Descriptive Statistics

Table 2 shows the descriptive statistics of the independent and dependent variables, and parameters in this study. In 2016, the percentage of households with MLTH was 11.4%. The absolute income average, which was the basis of the parameters, was 30,862 US dollars in 2015, 23,399 US dollars in 2016, and 19,060 US dollars in 2017. There was a significant difference. The EIRR was calculated as the difference between earned income in the years before and after 2016 (2015 to 2017). Results show that it declined by about 49% after MLTH. The dependent variable, CHE in 2017, was also increased to 22.3% from 19.2% in 2015. 

### 3.3. Mediating Effect of Earned Income Loss on the Relationship between MLTH and CHE

The results of the mediating effect model were as follows: Model 1 showed the direct effect of MLTH on CHE (Table 3). When MLTH occurred, the probability of incurring CHE was significantly higher (*p* < 0.01). In Model 2 (Table 4), the MLTH increased EIRR (*p* < 0.05). Model 3 examined the effect of MLTH and EIRR on CHE (Table 5). Although the effect of EIRR was controlled, MLTH increased the likelihood of CHE (*p* < 0.01), and even in the case of the EIRR, it had a positive effect while controlling MLTH (<0.01). As the results of the second and third stages, it can be regarded that there was a mediating effect because the EIRR showed a significant path coefficient in both stages. Since they had a significant effect under the control of each other, it can be seen that there is a partial mediating effect.

Bootstrapping was conducted to test whether EIRR had a mediating effect on the relationship between MLTH and CHE. The number of samples re-extracted for bootstrapping was 1000. The lower limit of the median effect coefficient obtained was 0.364, and the upper limit was 0.644, and therefore it did not contain a value of 0. Through this, it was confirmed that the EIRR mediates the effect of MLTH on CHE.

## 4. Discussion

This study contributes to the literature by giving another insight in interpreting the cause of CHE, focusing on income loss factors, other than the existing studies that interpreted the cause of CHE only as medical expenses. Additionally, it found that MLTH would increase both medical expenses and earned income loss. The fundamental purpose of this study was achieved by confirming that CHE can be significantly affected by MLTH, which affects income loss as well as medical expenses.

We analyzed whether MLTH affects the occurrence of CHE through the mediating effect of EIRR using the data from the KoWePS. For the analysis, we applied the three steps regression analyses suggested by Baron and Kenny [19]. Since the CHE indicator measures the burden of medical expenditures by households, the mediating effect of EIRR also needs to be examined in household units.

In the first stage, MLTH was significantly positively associated with the occurrence of CHE. Since the MLTH was in 2016 and CHE was in 2017, it should be considered that there is a time difference. MLTH implies that health condition is worsened to a greater extent than outpatient or short-term hospitalization, so it can be interpreted that medical expenses continue to occur in the next year as it continues to affect not only the year of hospitalization but also the increase in medical use afterward.

In the second stage, it was confirmed that the degree of EIRR increased when MLTH occurred. In the third stage, MLTH and EIRR were simultaneously put into the model and analyzed, all of which had a significant positive effect. To verify the mediating effect of earned income, we used bootstrapping. The 95% confidence interval of the bootstrap was from 0.364 to 0.644, which did not include 0, so it can confirm that the EIRR mediates the effects of MLTH on CHE.

From these results, we could figure out there is a possibility that MLTH could make earned income loss. This may be because the earned income is closely related to work. MLTH limits work hours inevitably, and it could be reflected in the pay. Furthermore, MLTH may lead to unemployment. Employers would not wait for workers who are hospitalized for too long. In this case, one could imagine the earned income may be zero, which is not the case, because the length of stay in hospital is not more than a year in general. Furthermore, people would find another job. However, even if they did, it would be at a lower wage compared with the previous job, and, therefore, there might still be a reduction of earned income [24]. Additionally, since MLTH in this study was measured by household unit occurrence, there may be financial strategies among household members to make up for the loss of earned income. For example, a household member’s cessation of income activities may force other members to search for jobs. However, even in this case, the wage level of a newly employed person is more likely to be lower than that of a previously employed person [24], so the effect of reducing earned income may still appear.

Previous studies have reported that CHE has a high reoccurrence rate [12,25,26,27] and affects poverty reduction and persistence [11,25,28]. According to Kwon [6], while medical expenses occur in the short term, the effect of income decline due to deterioration in health appeared in the long term. Recalling this result, persistent CHE reoccurrence and poverty may also be a phenomenon caused by the underlying health deterioration affecting income rather than the CHE occurrence itself. Therefore, it should be considered that CHE is not only caused by medical expenses but may arise from health affecting the decline in earned income.

Although it is important to focus on the medical expense coverage policy when considering the causes and solutions of CHE, the problem of income instability caused by deteriorating health should also be addressed. Therefore, a representative insurance system for income loss due to illness is essential in Korea. 

Many households deal with the burden of medical expenses through financial coping strategies, such as borrowing money [29]. Still, it is difficult to overcome the long-term loss of income without external help, such as assistance from a government or any social group. For example, when medical expenses are incurred, it is possible to borrow money from relatives and acquaintances; because there is compassion for the patient, the purpose of borrowing is clear, and it is a short-term expense. However, it becomes difficult to borrow money when one loses a job due to illness, because most people tend to lend money only when they have the possibility of getting it back. Additionally, in the long run, the cost of living is higher than medical expenses.

Since the CHE is the ratio of medical expenses to households’ ability to pay, it is important to recognize that it may occur due to income loss. Therefore, it is necessary to recognize that the burden of medical expenses can be reduced by guaranteeing income. Especially nowadays, there is a greater need for a health-related income security system with the re-emergence of infectious diseases such as new influenza viruses, Middle East respiratory syndrome, coronavirus worldwide, since infectious diseases risk other people, and so they are subject to quarantine. The quarantine, which is similar to MLTH, can lead directly to loss of income, especially for small business owners, daily workers, and specialty workers. If an infectious disease becomes widespread, consumption activity shrinks—decreasing income and increasing the number of bankrupt workers and businesses. There could even be an economic recession in one country and around the world. This pattern will have a more lethal effect in countries with much social security left to the private sector. Therefore, CHE should be considered in terms of income security in addition to medical cost coverage, and the introduction of sickness benefits in the public social security system is highly needed.

This study has two limitations. First, MLTH as the independent variable was defined as household with more than one member who use more than 7 days of hospitalization, so it was not possible to distinguish whether the household members who had been hospitalized were employed. However, even if nonworking patients are hospitalized, family members who are employed may stop working to care for them. Therefore, this study has value in that it included both employed and unemployed patients who had been hospitalized. Second, the difference in the standardized coefficients of MLTH between the first and the third stage of the analysis was small (0.078 and 0.07, respectively) (Table 3 and Table 5). The reasons are as follows: (1) In the case of the standardized regression coefficient, the difference in values tends to be smaller than that of the non-standardized regression coefficient. (2) The derived value may have been lower because the earned income was converted to a common log. When a variable such as income is converted into a log value and measured, the distribution of exponentially increasing costs become small [17]. (3) The denominator of CHE is gross household income, and the decrease in earned income, which is a parameter, is a small portion. Additionally, since the value decreased further in the process of deducting income between 2015 and 2017, it is highly likely that the regression coefficient was low. The fact that the difference in the regression coefficient values between models is small may seem to be a limitation, but the actual effect can be expected to be greater, and it is significant in that the mediating effect has appeared despite the condition, which has limitations as suggested above.

## 5. Conclusions

Previous studies have only focused on medical costs when interpreting the CHE indicator. However, it is also essential to recognize that the MLTH can have a negative effect on the household’s ability to pay, which comprises CHE. MLTH can be used as a proxy for a significant level of deterioration in health. Additionally, since the MLTH is directly linked to the discontinuation of work activities, it can affect income loss. Therefore, policymakers should consider ideas to ensure income security during illness, including sickness benefits in the public social security system.

## Figures and Tables

**Table 1:** Sociodemographic characteristics of the study subjects.

Variable		Division	Frequency	%
Householder characteristic	Gender	Man	1272	76.1
		Woman	399	23.8
	Age	≤39	531	31.7
		40–64	882	52.7
		≥65	258	15.4
	Marital status	Married	1106	66.1
		Not married	94	5.6
		Divorced, widowed, or separated	471	28.1
	Education level	University or higher	435	26.0
		Elementary school or less	797	47.7
		Middle school and high school	439	26.2
	Job type	Waged worker	785	46.9
		Employer or self-employed	346	20.7
		Unpaid family worker	50	2.9
		Unemployed	490	29.3
Household characteristic	No. of household members	1	318	19.0
		2	555	33.2
		3	382	22.8
		≥4	416	24.9
	Medical coverage type	Employee	1075	64.3
		Employer or self-employed	501	29.9
		Medical benefit recipient	95	5.6
	Private health insurance	No	1165	69.7
		Yes	506	30.2
	Disability	No	1589	95.0
		Yes	82	4.9
	Chronic disease	No	464	27.7
		Yes	1207	72.2

**Table 2:** Descriptive statistics of key variables.

Variable			Frequency (%)	Average (SD)
Independent variable	Mid-to-long-term hospitalization	Occurred	192 (11.4)	-
		Not Occurred	1479 (88.5)	
Parameter	Earned income (reduced)	2015	1671	30,862.7 (40,259.2) *
		2016		23,398.9 (25,839.1) *
		2017		19,060.8 (23,397.1) *
	EIRR	2015–2017	-	49.06 (36.9) **
Dependent variable	CHE of 2015	Occurred	321 (19.2)	-
		Not Occurred	1350 (80.8)	
	CHE of 2017	Occurred	374 (22.3)	-
		Not Occurred	1297 (77.7)	

Note: * unit = US dollar, ** unit = percentage (%), EIRR = earned income reduction rate.

**Table 3:** The effect of MLTH on CHE (Model 1).

Variable (Reference)		Step 1		
		B	S.E.	Beta
Mid-to-long-term hospitalization	Occurrence	0.492	0.182	0.078 ***
Gender (man)	Woman	0.014	0.227	0.003
Ages (≤39)	40–64	−0.112	0.158	−0.028
	≥65	0.335	0.183	0.060 *
Marital status (married)	Not married	−0.715	0.378	−0.082 *
	Divorced, widowed, or separated	−0.254	0.230	−0.057
No. of household members (1)	2	0.106	0.227	0.025
	3	−0.291	0.262	−0.062
	≥4	−0.504	0.290	−0.112 *
Education level (university or higher)	Elementary school or less	0.031	0.181	0.008
	Middle and high school	0.446	0.216	0.098 **
Medical coverage type (self-employed or employees)	Uninsured or self-payer	−0.040	0.143	−0.009
	Medical benefit recipient	−1.436	0.373	−0.166 ***
Job type (waged worker)	Employer or self-employed	0.252	0.180	0.051
	Unpaid family worker	−0.370	0.402	−0.031
	Unemployed	0.450	0.166	0.102 ***
Private health insurance (no)	Yes	0.292	0.161	0.067 *
Disability (no)	Yes	0.201	0.287	0.022
Chronic disease (no)	Yes	0.768	0.180	0.171 ***
Constant term		−2.064	0.303	−2.064 ***

Note: 1-step logit regression analysis: The effect of MLTH on CHE. The independent variable, MLTH, is based on the year 2015 and the dependent variable, CHE, is based on the year 2017. B: Unnormalized regression coefficient, S.E.: Standard error of unnormalized regression coefficient, Beta: Standardized regression coefficient. * *p* < 0.1, ** *p* < 0.05, *** *p* < 0.01. In the results of additional analysis, even after controlling for MLTH in the 2017, the previous year’s MLTH had a significant effect.

**Table 4:** The effect of MLTH on the loss of earned income (Model 2).

Variable (Reference)		Step 2		
		B	S.E.	Beta
Mid-to-long-term hospitalization	Occurrence	0.196	0.083	0.050 **
Gender (Man)	Woman	0.095	0.091	0.032
Age (≤39)	40–64	−0.158	0.064	−0.063 **
	≥65	0.068	0.088	0.019
Marital status (married)	Not married	−0.015	0.131	−0.002
	Divorced, widowed, separated	0.015	0.093	0.005
No. of household members (1)	2	−0.076	0.094	−0.028
	3	−0.228	0.105	−0.078 **
	≥4	−0.491	0.115	−0.175 ***
Education level (university or higher)	Elementary school or less	−0.031	0.068	−0.012
	Middle school and high school	−0.052	0.090	−0.018
Medical coverage type (self-employed or employees)	Uninsured or self-payer	0.229	0.061	0.084 ***
	Medical benefit recipient	0.221	0.124	0.041 *
Job type (waged worker)	Employer or self-employed	0.988	0.075	0.322 ***
	Unpaid family worker	0.261	0.163	0.035
	Unemployed	0.869	0.072	0.318 ***
Private health insurance (No)	Yes	0.190	0.071	0.070 ***
Disability (No)	Yes	0.216	0.127	0.037 *
Chronic disease (No)	Yes	0.114	0.062	0.041 *
Constant term		2.960	0.116	2.960 ***

Note: 2-step linear regression analysis: The effect of MLTH on the decrease in earned income. The decrease in earned income is the difference between the income in the year 2015 and the income in the year 2017 when a MLTH occurred in the year 2016. Independent variables are based on the year 2016, parameters and dependent variables are based on the year 2017. B: Unnormalized regression coefficient, S.E.: Standard error of unnormalized regression coefficient, Beta: Standardized regression coefficient, * *p* < 0.1, ** *p* < 0.05, *** *p* < 0.01.

**Table 5:** The effect of income decline on the relationship between MLTH and CHE.

Variable (Reference)		Step 3		
		B	S.E.	Beta
Mid-to-long-term hospitalization	Occurrence	0.449	0.183	0.070 **
Earned income	Continuous variable	0.316	0.071	0.193 ***
Gender (man)	Women	−0.032	0.227	−0.007
Age (≤39)	40–64	−0.068	0.160	−0.017
	≥65	0.333	0.184	0.059 *
Marital status (married)	Not married	−0.691	0.380	−0.078 *
	Divorced, widowed, or separated	−0.241	0.230	−0.053
No of household members (1)	2	0.118	0.227	0.027
	3	−0.227	0.263	−0.047
	≥4	−0.381	0.292	−0.083
Education level (university or higher)	Elementary school or less	0.054	0.182	0.013
	Middle school and high school	0.480	0.217	0.104 **
Medical coverage type (self-employed or employees)	Uninsured or self-payer	−0.092	0.145	−0.021
	Medical benefit recipient	−1.494	0.373	−0.170 ***
Job type (waged worker)	Employer or self-employed	−0.036	0.190	−0.007
	Unpaid family worker	−0.491	0.407	−0.041
	Unemployed	0.180	0.177	0.040
Private health insurance (no)	Yes	0.222	0.163	0.050
Disability (no)	Yes	0.151	0.287	0.016
Chronic disease (no)	Yes	0.732	0.181	0.161 ***
Constant term		−3.044	0.382	−3.044 ***

Note: 3-step logit regression analysis: The effect of MLTH on CHE. Independent variables are based on the year 2016, and parameters and dependent variables are based on the year 2017. B: Unnormalized regression coefficient, S.E.: Standard error of unnormalized regression coefficient, Beta: Standardized regression coefficient. * *p* < 0.1, ** *p* < 0.05, *** *p* < 0.01.

## Data Availability

The data that support the findings of this study are available from KoWePS but restrictions apply to the availability of these data, which were used under license for the current study, and so are not publicly available. However, data are available from the authors upon reasonable request and with permission of KoWePS.

## Abbreviations

CHE	Catastrophic health expenditure
EIRR	Earned income reduction rate
KoWePS	Korean Welfare Panel Study
MLTH	Mid-to-long-term hospitalization
